# TMEM16A Plays an Insignificant Role in Myocardium Remodeling but May Promote Angiogenesis of Heart During Pressure-overload

**DOI:** 10.3389/fphys.2022.897619

**Published:** 2022-05-31

**Authors:** Yaofang Zhang, Lingyu Ye, Dayue Darrel Duan, Hong Yang, Tonghui Ma

**Affiliations:** ^1^ College of Basic Medical Sciences, Dalian Medical University, Dalian, China; ^2^ The Laboratory of Cardiovascular Phenomics, Department of Pharmacology, University of Nevada School of Medicine, Reno, NV, United States; ^3^ Liaoning Provincial Key Laboratory of Biotechnology and Drug Discovery, School of Life Sciences, Liaoning Normal University, Dalian, China

**Keywords:** TMEM16A, cardiac hypertrophy, heart failure, stress, angiogenesis

## Abstract

**Background:** Cardiac hypertrophy (CH) occurs with an increase in myocardium mass as an adaptive compensation to increased stress. Prolonged CH causes decompensated heart failure (HF). Enhanced angiogenesis by vascular endothelial growth factor (VEGF) is observed in hypertrophied hearts; impaired angiogenesis by angiotensin II (AngII) is observed in failing hearts. Angiogenesis is executed by vascular endothelial cells (ECs). Abnormal Ca^2+^ homeostasis is a hallmark feature of hypertrophied and failing hearts. Ca^2+^-activated chloride channel transmembrane protein 16A (TMEM16A) is expressed in cardiomyocytes and ECs but its role in heart under stress remains unknown.

**Methods:** Pressure-overload-induced CH and HF mouse models were established. Echocardiography was performed to evaluate cardiac parameters. Quantitative real-time PCR, traditional and simple western assays were used to quantify molecular expression. Whole-cell patch-clamp experiments were used to detect TMEM16A current (I_TMEM16A_) and action potential duration (APD) of cardiomyocytes. VEGF and AngII were used separately in ECs culture to simulate enhanced or impaired angiogenesis, respectively. TMEM16A low-expressed and over-expressed ECs were obtained by siRNA or lentivirus transfection. Wound healing, tube formation and ECs spheroids sprouting assays were performed to assess migration and angiogenesis.

**Results:** Neither TMEM16A molecular expression levels nor whole-cell I_TMEM16A_ density varied significantly during the development of CH and HF. I_TMEM16A_ comprises transient outward current, but doesn’t account for APD prolongation in hypertrophied or failing cardiomyocytes. In cultured ECs, TMEM16A knockdown inhibited migration and angiogenesis, TMEM16A overexpression showed opposite result. Promotion of migration and angiogenesis by VEGF was decreased in TMEM16A low-expressed ECs but was increased in TMEM16A over-expressed ECs. Inhibition of migration and angiogenesis by AngII was enhanced in TMEM16A low-expressed ECs but was attenuated in TMEM16A over-expressed ECs.

**Conclusion:** TMEM16A contributes insignificantly in myocardium remodeling during pressure-overload. TMEM16A is a positive regulator of migration and angiogenesis under normal condition or simulated stress. TMEM16A may become a new target for upregulation of angiogenesis in ischemic disorders like ischemic heart disease.

## 1 Introduction

TMEM16A was identified as a Ca^2+^-activated chloride channel (CACC) in 2008. It is wildly distributed and is involved in various physiological and pathological events (Oh and Jung, 2016). TMEM16A is expressed and induces Ca^2+^-activated chloride current (I_Cl.Ca_) in human atrial cardiac fibroblasts cells (El Chemaly et al., 2014) and mouse ventricular myocytes (Ye et al., 2015). I_Cl.Ca_ exists in several cardiac cell types, it takes part in the formation of transient outward current (I_to_) or transient inward current (I_ti_), and may contribute to delayed afterdepolarizations, early afterdepolarizations, APD, arrhythmia and cardiac ischemia. Under physiological or pathological conditions, I_Cl.Ca_ is elicited by increment of intracellular Ca^2+^ concentration in cardiac cells. Intracellular Ca^2+^ concentration is determined by Ca^2+^ influx via voltage-gated Ca^2+^ channels and Ca^2+^ release from sarcoplasmic reticulum (SR), i.e. I_Cl.Ca_ is related with Ca^2+^ homeostasis (Tseng and Hoffman, 1989; Zygmunt and Gibbson, 1991; Zygmunt, 1994; Collier et al., 1996; Verkerk et al., 2000; Xu et al., 2002; Ye et al., 2015; Kanaporis and Blatter, 2016; Hegyi et al., 2017). Despite the multiple causes and clinical manifestations of CH and HF, abnormal Ca^2+^ homeostasis is a hallmark feature of hypertrophied and failing hearts (Houser et al., 2000). The role of I_Cl.Ca_ in HF was rarely studied and appeared controversial. Verkerk et al. found that HF per se did not alter I_Cl.Ca_ density in rabbit, and I_Cl.Ca_ may be absent during delayed afterdepolarizations in nonfailing human ventricular cells (Verkerk et al., 2001). But Pu et al. reported that I_Cl.Ca_ density decreased significantly in failing canine cardiomyocytes, which may contribute to the prolongation of APD in failing heart (Pu et al., 2006). No investigation declares the role of TMEM16A in CH and HF.

The heart responds to stress such as pressure-overload with an increase in myocardium mass (myocardium remodeling) to maintain cardiac function. This hypertrophic response is initially considered adaptive and involves an increase in microvascular density. Secretion of angiogenic growth factors such as VEGF from hypertrophied cardiomyocytes is responsible for enhanced angiogenesis (Sano et al., 2007; Oka et al., 2014). However, with sustained stress, the heart eventually transitions into a maladaptive phase with suppression of microvascular density and impaired angiogenesis observe. Reduced VEGF, as well as overproduced AngII, the main component of renin-angiotensin system activated in conditions of increased stress of cardiomyocytes, are mostly responsible for dysregulation angiogenesis (Schunkert et al., 1990; Masuda et al., 2012; Oka et al., 2014). Angiogenesis is the formation of new blood vessel branches which relies on vascular ECs. Human umbilical vein endothelial cells (HUVECs) were commonly used for angiogenesis studies (Nowak-Sliwinska et al., 2018). TMEM16A is expressed in several EC types and displays dissimilar even paradoxical functions (Wu et al., 2014; Ma et al., 2017; Ayed et al., 2018; Liu et al., 2019; Suzuki et al., 2020; Ma et al., 2021). The relationship between TMEM16A and angiogenesis under stress is unknown. In fact, there was no report about TMEM16A and angiogenesis existed yet.

In this study, we sought to investigate the role of TMEM16A in remodeling of myocardium during pressure-overload and the role of TMEM16A in angiogenesis under stress.

## 2 Materials and Methods

### 2.1 Medication

All chemicals without annotation in brackets were purchased from Sigma-Aldrich in this study.

### 2.2 Establishment of Animal Models

140 male C57BL/6 (wild type, 8–10 weeks old) mice were purchased from Jackson Laboratory and were housed 1 week for adaption before experiments. The feeding and surgical operations of mice were carried out in SPF conditions. All animal protocols were approved by the Animal Care and Use Committee of University of Nevada, Reno. This investigation conforms to the Guide for the Care and Use of Laboratory Animals (NIH).

Pressure-overload-induced CH and HF mouse models were developed through a modified aortic banding (AB) surgery (Rockman et al., 1991). Briefly, mouse was intubated and ventilated by a mouse ventilator (Harvard Apparatus) with 2% isoflurane contained air. A small midline chest incision was made to expose the aorta and branching arteries, and aortic constriction was performed on position between brachiocephalic trunk artery and left carotid artery by tying a 8–0 silk suture ligature against a 27-gauge needle to yield a narrowing 0.4 mm in diameter when the needle was removed. Sham control mice underwent chest incision surgery but the aortas were not narrowed.

### 2.3 Echocardiography

To evaluate the parameters of mouse heart, echocardiographic measurements were performed using Vevo^®^ 2100 imaging system (Visualsonics) with MS-550D ultrasound transducer according to the manuals every week after surgery. In brief, mouse was anesthetized with isoflurane (2%) and fixed to a warming platform in a supine position. The heart rate monitored by electrocardiogram was kept from 450 to 500 per minute. Both B-mode and M-mode of parasternal long-axis and short-axis views of left ventricle (LV) were recorded at a frame rate of 200 Hz. All data analysis was done using Vevo^®^ LAB desktop software.

### 2.4 Left Ventricular Myocytes Isolation

Left ventricular myocytes (LVMs) were isolated by an enzymatic dispersion technique (Xu et al., 2002). In brief, mouse was killed by cervical dislocation, and heart was rapidly excised. Aorta was cannulated to the syringe tip of Langendorff apparatus, and retrograde perfusion of the aorta with various buffer solutions gassed with 100% O_2_ and maintained at 37°C was performed. Heart was firstly perfused with Tyrode solution (in mM: 125 NaCl, five MgCl_2_, 4.5 KCl, 10 HEPES, one NaH_2_PO_4_, five pyruvic acid, 20 taurine, 10 glucose, 1.5 CaCl_2_, pH 7.4 with NaOH) until the blood was washed, then perfused with Ca^2+^-free Tyrode solution for 5 min, followed by Ca^2+^-free Tyrode solution containing 0.4 mg/mL collagenase type II (Wirthington) and 1 mg/mL albumin for 10–15 min. LV was separated and soaked in Kreb’s solution (in mM: 20 KCl, one MgCl_2_, 10 KH_2_PO_4_, 70 l-glutamic acid, 10 hydroxybutyric acid, 10 taurine, 10 EGTA, 10 glucose, 1 mg/mL albumin, pH 7.4 with KOH). LVMs can be dispersed by suction the solution repeatedly with straw.

### 2.5 Whole-Cell Patch-Clamp Recordings

Rod-shaped LVMs exhibited clear cross striations were used in patch-clamp experiments which were carried out at room temperature (20–25°C) using an Axopatch 200B amplifier (Axon Instruments). Data were filtered at 1 kHz and sampled at 5 kHz. Borosilicate glass electrodes had 3–5 MΩ tip resistances when filled with pipette solution (in mM: 110 potassium aspartate, 20 KCl, one MgCl_2_, five ATP-Mg, 0.1 GTP, 10 HEPES, five Na_2_-phosphocreatine, 0.05 EGTA, pH 7.4 with KOH). For action potential (AP) recordings, the bath solution contained (in mM) 126 NaCl, two CaCl_2_, 5.4 KCl, 0.8 MgCl_2_, 0.33 NaH_2_PO_4_, 10 dextrose and 10 HEPES (pH 7.4 with NaOH). For whole-cell current recordings, Na^+^ in bath solution was replaced with NMDG to block Na^+^-Ca^2+^ exchange current. The equilibrium potential for Cl^−^ (E_Cl_) was -48 mV. Pipette resistance was compensated. Cell membrane capacitance (C_m_) was determined by integrating the capacity transient elicited by a 5 mV hyperpolarizing pulse from a holding potential of -50 mV. C_m_ and series resistance were compensated as much as possible. LVMs with resting membrane potentials between -60 and -80 mV were used.

A phenomenon known as “rundown” is the gradually disappearance of I_Ca_ when cells are dialyzed or perfused. Cardiac cell desires 15–30 s intervals between depolarization pulses to allow recovery of ionic currents and intracellular Ca^2+^ metabolism, but rundown is accelerated by perfusion or pulses that elicit I_Ca_ (Zygmunt and Gibbson, 1991). In addition, depolarization sometimes causes contraction of cardiac cell and leads to “seal-lost”. Here, in order to save time and to reduce depolarizing stimuli, whole-cell currents were elicited by depolarizing voltage steps (200 ms) from -20 to +60 mV in 20 mV increments from a holding potential of -50 mV to determin I-V relations. When comparing I_TMEM16A_ densities of LVMs between sham and AB mice, currents were activated by voltage steps from a holding potential of -50 to +60 mV. TMEM16A-specific potent inhibitor T16A_inh_-A01, and Anti-TMEM16A antibody (ab53212, Abcam) with synthetic peptide that detects the extracellular domain amino acids 628–731 of TMEM16A (including the predicted pore-forming domain) were used separately as I_TMEM16A_ inhibitors. All peak current values were normalized to C_m_ and reported as current densities (pA/pF). APs were elicited by 5 ms current pulses (500 pA) applied via the patch pipette at stimulus frequencies of 1 Hz.

### 2.6 HUVECs Isolation and Culture

HUVECs were isolated and cultured by a modified sterile technique described previously (Jaffe et al., 1973). The experiments were approved by the medical research ethics committee of Dalian Medical University and conformed to the principles expressed in the Declaration of Helsinki. Briefly, umbilical veins were perfused by D-Hanks buffer (in g/L: 8.0 NaCl, 0.4 KCl, 0.06 KH_2_PO_4_, 0.35 NaHCO_3_), and HUVECs were harvested from the umbilical veins digested by 0.25% trypsin (Beyotime) with 0.02% EDTA. Then cells were cultured in ECM medium (sciencell 1001, Solarbio) containing 5% fetal bovine serum, 1% ECGs, 0.1% Savelt, 100 U/mL penicillin and 100 mg/mL streptomycin at 37°C, 5% CO_2_ humidified atmosphere. Cells between passages three and five were used in this study.

### 2.7 siRNA Transfection

Three kinds of siRNA (#1, #2 and #3) against human TMEM16A were designed and constructed by GenePharma (China). siRNA transfection of HUVECs was performed with Lipofectamine 2000 (Invitrogen) according to the manufacturer’s protocol. Negative siRNA (GenePharma) was used as negative control (siRNA^NC^). The sequences of siRNA were as follows: siRNA #1, sense (5′- CCG​GAG​CAC​GAU​UGU​CUA​UTT -3′), antisense (5′- AUA​GAC​AAU​CGU​GCU​CCG​GTT -3′); siRNA #2, sense (5′- GGA​AAC​AGA​UGC​GAC​UCA​ATT -3′), antisense (5′- UUG​AGU​CGC​AUC​UGU​UUC​CTT -3′); siRNA #3, sense (5′- GCU​GUC​AAG​GAU​CAU​CCU​ATT -3′), antisense (5′- UAG​GAU​GAU​CCU​UGA​CAG​CTT -3′); siRNA^NC^, sense (5′- UUC​UCC​GAA​CGU​GUC​ACG​UTT -3′), antisense (5′- ACG​UGA​CAC​GUU​CGG​AGA​ATT -3′). Briefly, cells at 70% confluent were starved with serum-free ECM for 1 h, and treated with siRNA/lipofectamin Opti MEM (Invitrogen) for 4–6 h. After cultured with complete ECM for 72 h, HUVECs were harvested for the following experiments.

### 2.8 Lentivirus Transfection

Lentivirus which can induce mouse TMEM16A overexpression (TM^OE^) was designed and constructed by Genechem (China). Lentivirus transfection of HUVECs was performed with polybrene (5 µg/mL) according to the manufacturer’s protocol. Negative lentivirus (Genechem) was used as negative control (TM^NC^). In brief, cells at 30–50% confluent were treated with lentivirus/polybrene complete ECM for 8–12 h and then cultured with complete ECM for 72 h. HUVECs were harvested for the following experiments.

### 2.9 Quantitative Real-Time PCR (qRT-PCR)

Total RNAs were extracted from mice LV or HUVECs using TRIzol reagent (Invitrogen), qualified and quantified by a Nanodrop 2000 spectrophotometer. Equal amounts (0.5 µg) of total RNAs were reverse transcribed with SuperScript IV RT reagent Kit (Invitrogen).

The expression levels of mRNAs in LV of mice were detected on 7900 HT Real-time PCR System by RT^2^ qPCR Primer Assays (Qiagen) according to the manufacturer’s protocol. The relative expression level of TMEM16A mRNA was calculated using the 2^-∆∆Ct^ method with GAPDH mRNA as a reference. Primers for mTMEM16A (Catalog no 330001 PPM26917B) and mGAPDH (Catalog no 330001 PPM02946E) were purchased from Qiagen.

The expression levels of mRNAs in HUVECs were detected on 7900 HT Real-time PCR System with TransStart Top Green qPCR SuperMix (TransGen Biotech) according to the manufacturer’s protocol. The relative expression level of TMEM16A mRNA was calculated using the 2^-∆∆Ct^ method with GAPDH mRNA as a reference. The sequences of primers were as follows: hTMEM16A, forward (5′- TGA​AAC​TGA​AGA​TGC​CGA​CG -3′), reverse (5′- AGG​AGA​GTC​TCT​TCA​TGG​TCT​G -3′); mTMEM16A, forward (5′- GAG​GCC​AGT​AGC​CAT​CAG​AG -3′), reverse (5′- GAG​AGC​GTG​TGA​TTG​ACG​AA -3′); hGAPDH, forward (5′- AGG​GCT​GCT​TTT​AAC​TCT​GGT -3′), reverse (5′- CCC​CAC​TTG​ATT​TTG​GAG​GGA -3′).

### 2.10 Western Blot

Total membrane protein was extracted from mice LV using Plasma Membrane Protein Extraction Kit (101 Bio) according to the manufacturer’s protocol. Total protein was extracted from HUVECs using RIPA lysis buffer (Solarbio) according to the manufacturer’s protocol.

#### 2.10.1 Traditional Western Blot

Briefly, total protein was separated on 10% SDS-PAGE, transferred onto a PVDF membrane and blotted with the following primary antibodies separately: Anti-TMEM16A antibody (ACL-011, Alomone labs) for protein extracted from mouse heart, Anti-TMEM16A antibody (BA3464-2, Boster) for endogenous human TMEM16A protein in HUVECs, Anti-TMEM16A antibody (ab53212, Abcam) for overexpressed mouse TMEM16A protein induced by lentivirus in HUVECs, and Anti-β-actin antibody (CST). After incubated with HRP-conjugated secondary antibodies, bands were detected using enhanced chemiluminescence (Invitrogen) and quantified by scanning densitometry (Bio-Rad Laboratories).

#### 2.10.2 Simple Western Blot

Simple western blot was performed as described (Chen et al., 2013) using Wes kit (PS-MK14 and PS-MK15, Protein Simple) on Wes System according to the instructions. The primary antibodies used were: Anti-TMEM16A antibody (ab53212, Abcam) and Anti-GAPDH antibody (Santa Cruz).

### 2.11 Wound Healing Assay

Briefly, HUVECs were incubated to grow into full confluence on 12-well plates. A wound was made in the cell monolayer in each well using a pipette tip. Serum free ECM with vehicle (PBS) or 30 ng/mL VEGF, or 1 μM AngII was added. Images of the wounds were taken under microscope after 3 h, 6 h and 12 h of incubation. Wound area was measured using ImageJ software and wound closure percentage was calculated to assess endothelial cell migration.

### 2.12 Tube Formation Assay

Tube formation assay was performed by a modified technique as described (Shi et al., 2013). In brief, HUVECs (2 × 10^4^ in 50 μL ECM with 0.1% BSA) were seeded on Ibitreat angiogenesis slides (Ibidi, Martinsried) pre-coated with 10 μL Matrigel. After incubation with serum free ECM with vehicle (PBS) or 30 ng/mL VEGF, or 1 μM AngII for 6 h, images were taken under microscope. Total branches length was quantified using ImageJ software to assess tube formation.

### 2.13 Endothelial Cell Spheroids Sprouting Assay

Endothelial cell spheroids sprouting assay was performed by a modified technique as described (Tang et al., 2016). Briefly, cell spheroids were generated overnight in hanging-drop culture consisting of 400 HUVECs in ECM and 20% methylcellulose. Spheroids were harvested and embedded in collagen type I gels (1 × M199 medium (Gibco), 1500 μg/mL collagen type I (BD Biosciences), 0.22% NaHCO_3_, pH 7.4 with NaOH). Then spheroids-containing gel was rapidly transferred into a 24-well plate and allowed to polymerize. Equal volume of 2 × MCDB medium (Gibco) with vehicle (PBS) or 60 ng/mL VEGF, or 2 μM AngII was added on top of the gel. After incubation for 24 h, images were taken under microscope, and total capillary-like sprouts length per spheroid was quantified using ImageJ software to assess capillary sprouting.

### 2.14 Statistics

Data were presented as mean ± SEM and analyzed by GraphPad Prism software. Statistical significance was determined by Student’s *t*-test or by one-way ANOVA followed by Bonferroni posttest, or by two-way ANOVA followed by a *post hoc* test (Bonferonni) for multiple comparisons. In some instances, sample-matched repeated measures (RM) ANOVA was used, as appropriate. *p* < 0.05 was considered statistically significant.

## 3 Results

### 3.1 Echocardiographic Characteristics of Mice

To evaluate the development of CH and HF, echocardiographic measurements were performed weekly until 12 weeks after surgery. As shown in Supplementary Figure S1, compared with sham control, AB mice showed significant increases in LV wall thickness, LV mass and LV mass index 1 week or 3 weeks postoperatively, suggesting that there was a compensatory increase in LV muscle responding to pressure-overload and CH occurred. From 1 to 12 weeks after surgery, both LV mass and LV mass index in AB mice increased continuously with time, and were significantly higher than those in sham group, indicating CH developed continuously. There was a slight decrease in dimension of the LV cavity in AB group at first; from 4th week, LV cavity dimension continued increasing and was significantly larger than sham group from 7th week, suggesting pathologic growth of the myocardium induced concentric remodeling of the ventricle transitioned to an eccentric remodeling (dilation). The average value of LV wall thickness in AB group reached a maximum at 8th week, and then decreased in the next few weeks. As shown in Supplementary Figure S2, in AB group, both LV short-axis shortening fraction and ejection fraction significantly decreased from 3th week, suggesting that LV systolic function significantly decreased. The LV chamber enlargement with loss of wall as well as destroyed systolic function are clinical features of HF.

### 3.2 TMEM16A Molecular Expression Levels in LV

**FIGURE 1:**
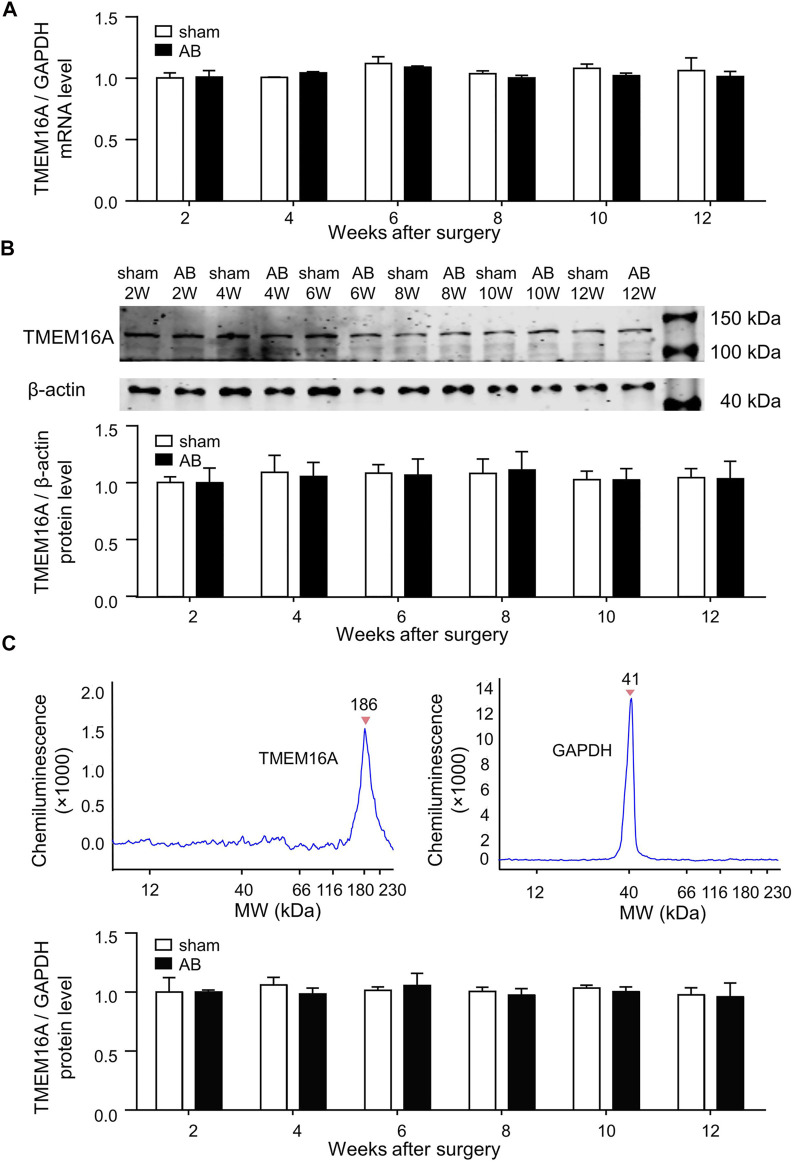
Molecular expression levels of TMEM16A in LV. **(A)** TMEM16A mRNA expression level in LV (*n* = 3 mice in each group per time point, RM two-way ANOVA). **(B)** Traditional western blot results of TMEM16A protein expression level in LV (*n* = 3 mice in each group per time point, RM two-way ANOVA). **(C)** Simple western blot results of TMEM16A protein expression level in LV (*n* = 3 mice in each group per time point, RM two-way ANOVA).

There was no significant difference in TMEM16A mRNA expression level or TMEM16A protein expression level in LV between sham and AB mice within 12 weeks after surgery (Figure 1). The molecular weight (MW) of TMEM16A is usually supposed to be 114 kDa, but in simple western blot assay, the peak with MW of 186 kDa was considered as TMEM16A (Figure 1C). It is reasonable according to the following facts. 1) In simple western assay, LV lysates mixed with different antibodies were separately loaded in different capillaries. Only one peak (186 kDa) emerged in the lane mixed with Anti-TMEM16A antibody, no peak existed in lane without antibody mixing. 2) The MW values marked in simple western assay may be higher than real values, since the peak represented for GAPDH here was marked 41 kDa, however 36 kDa was generally reported as the MW of GAPDH. 3) The MW of mTMEM16A overexpressed in HUVECs is nearly 180 kDa (Figure 5B). 4 Two MW values (85 kDa and 146 kDa) of TMEM16A exist in colon ECs from guinea pigs (He et al., 2011). The MW of TMEM16A in neonatal mice cardiac vascular ECs is between 130 and 170 kDa (Wu et al., 2014). 5) TMEM16A has different splicing isoforms, has species-specific and tissue-specific characteristics, and has post-translational modifications such as glycosylation as a functional membrane protein.

### 3.3 Whole-Cell I_to_ in LVMs

**FIGURE 2:**
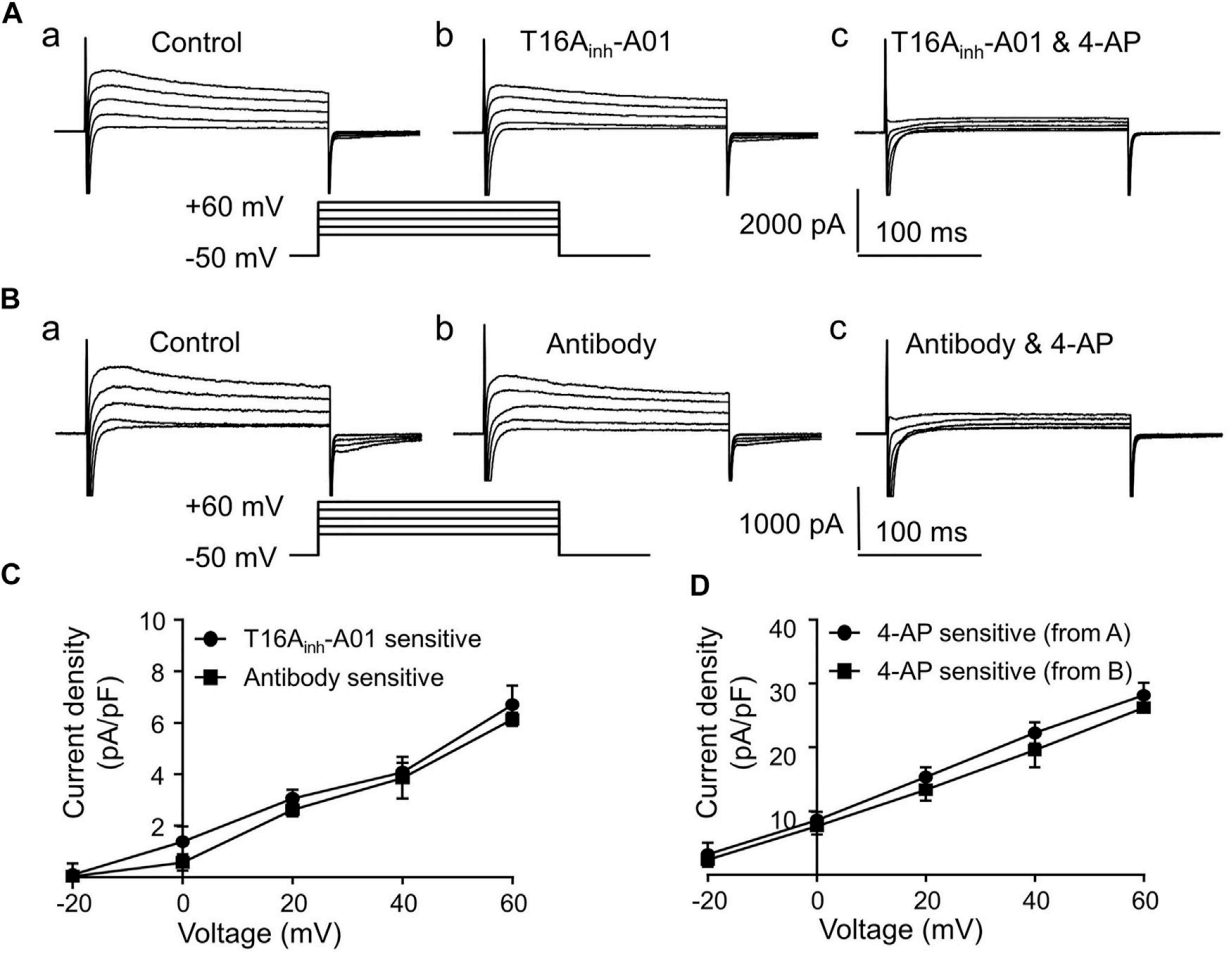
I_to_ in LVMs. **(A)** Recordings show the effects of T16A_inh_-A01 (30 μM) or 4-AP (5 mM) on I_to_ in LVMs. **(B)** Recordings show the effects of Anti-TMEM16A antibody (1:200) or 4-AP (5 mM) on I_to_ in LVMs. **(C)** I-V relations of T16A_inh_-A01 sensitive current and Anti-TMEM16A antibody sensitive current (*n* = 4 cells in each group, two-way ANOVA). **(D)** I-V relation of 4-AP sensitive current (*n* = 4 cells in each group, two-way ANOVA).

In this study, whole-cell I_to_-voltage (I-V) relations were determined using a voltage-clamp protocol (shown in the inset of Figure 2) to save time and to reduce depolarizing stimuli. In cardiac cells, I_to_ responsible for repolarization during phase one of AP is composed of I_Cl.Ca_ and 4-aminopyridine (4-AP) sensitive I_K_. As shown in Figure 2A, I_to_ were partly inhibited after adding T16A_inh_-A01 (30 μM), and were greatly inhibited after adding T16A_inh_-A01 along with 4-AP (5 mM) to the bath solution. The inhibition of I_to_ by Anti-TMEM16A antibody (1:200, adding extracellular) was similar to that by T16A_inh_-A01 (Figures 2A–C). Both T16A_inh_-A01 sensitive current and Antibody sensitive current were voltage-dependent, and their densities were much smaller than 4-AP sensitive current densities under the same depolarizing voltage (Figures 2C,D).

### 3.4 I_TMEM16A_ in LVMs

**FIGURE 3:**
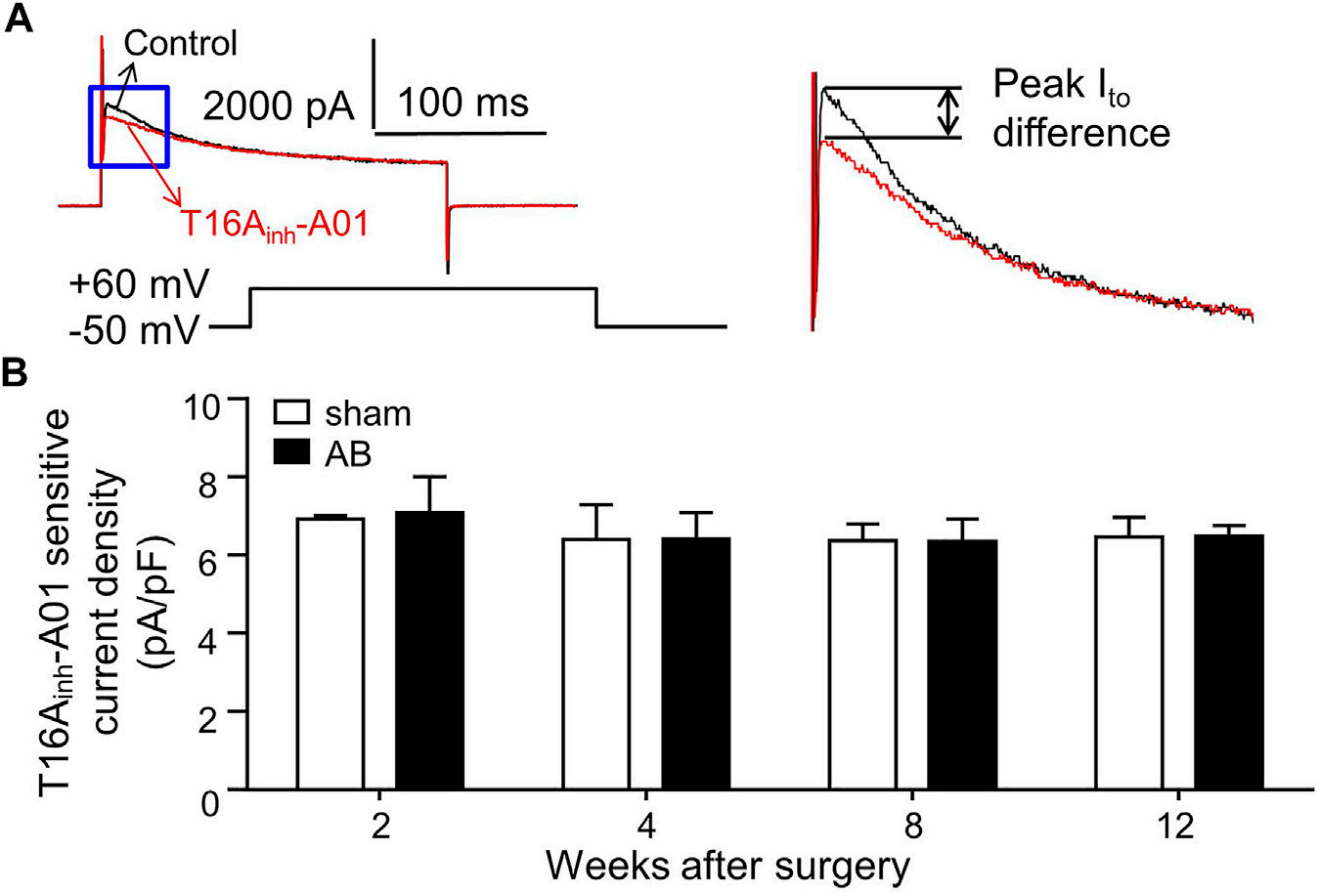
I_TMEM16A_ in LVMs. **(A)** Superimposed current tracings were recorded from the same cell before (control) and after exposure to 30 μM T16A_inh_-A01. Right panel shows expanded traces in the blue box of left panel. **(B)** Bar graphs show average T16A_inh_-A01 sensitive current densities in LVMs (*n* = 7—8 cells from three mice in each group per time point, RM two-way ANOVA).

As shown in Figure 3A, the superimposed current tracings were activated by voltage steps from a holding potential of -50 to +60 mV in the absence (control) and presence of T16A_inh_-A01 (30 μM) in the same cell. The peak I_to_ difference was measured as the T16A_inh_-A01 sensitive current (I_TMEM16A_) value. The I_TMEM16A_ densities were not significantly different in LVMs isolated from sham or AB mice within 12 weeks after surgery (Figure 3B).

### 3.5 AP in LVMs

**FIGURE 4:**
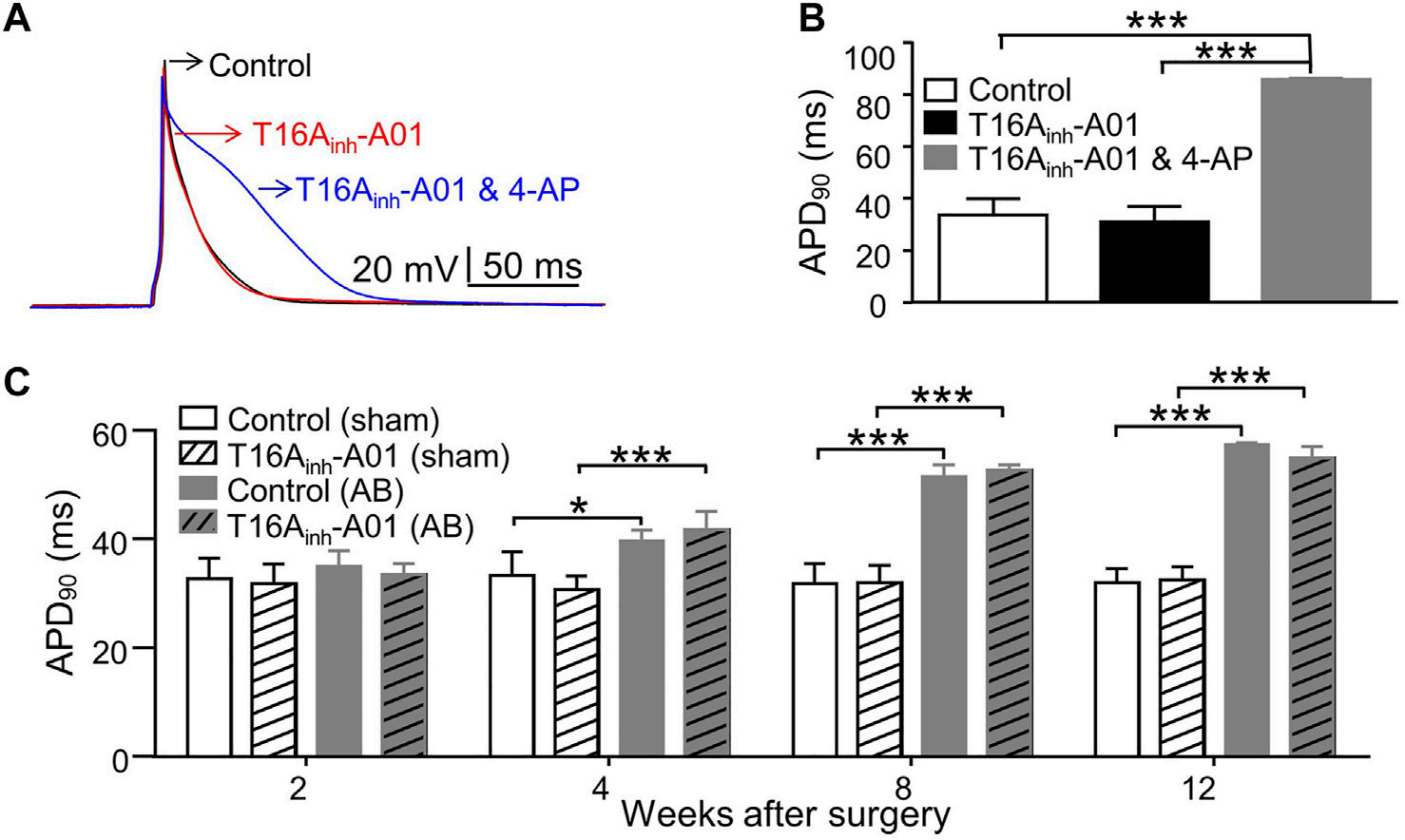
AP in LVMs. **(A)** Superimposed APs were recorded from the same cell before (control), after exposure to T16A_inh_-A01 (30 μM), and after exposure to T16A_inh_-A01 (30 μM) and 4-AP (5 mM). **(B)** Bar graphs show average APD_90_ in LVMs under conditions described above (*n* = 4 cells, ***p < 0.001, one-way ANOVA). **(C)** Bar graphs show average APD_90_ in LVMs of mice after surgery (n = 7—8 cells from three mice in each group per time point, *p < 0.05 and ***p < 0.001, RM two-way ANOVA).

Inhibition of TMEM16A by T16A_inh_-A01 (30 μM) didn’t significantly alter the AP shape or APD_90_, whereas inhibition of K^+^ channels by 4-AP (5 mM) significantly prolonged APD_90_ in LVMs (Figures 4A,B). LVMs of AB mice showed significant prolongation of APD_90_ 4 weeks postoperatively compared with sham LVMs (Figure 4C). TMEM16A inhibition by T16A_inh_-A01 insignificantly influenced the APD_90_ in LVMs of sham or AB mice within 12 weeks after surgery (Figure 4C).

### 3.6 TMEM16A Molecular Expression Levels in HUVECs

**FIGURE 5:**
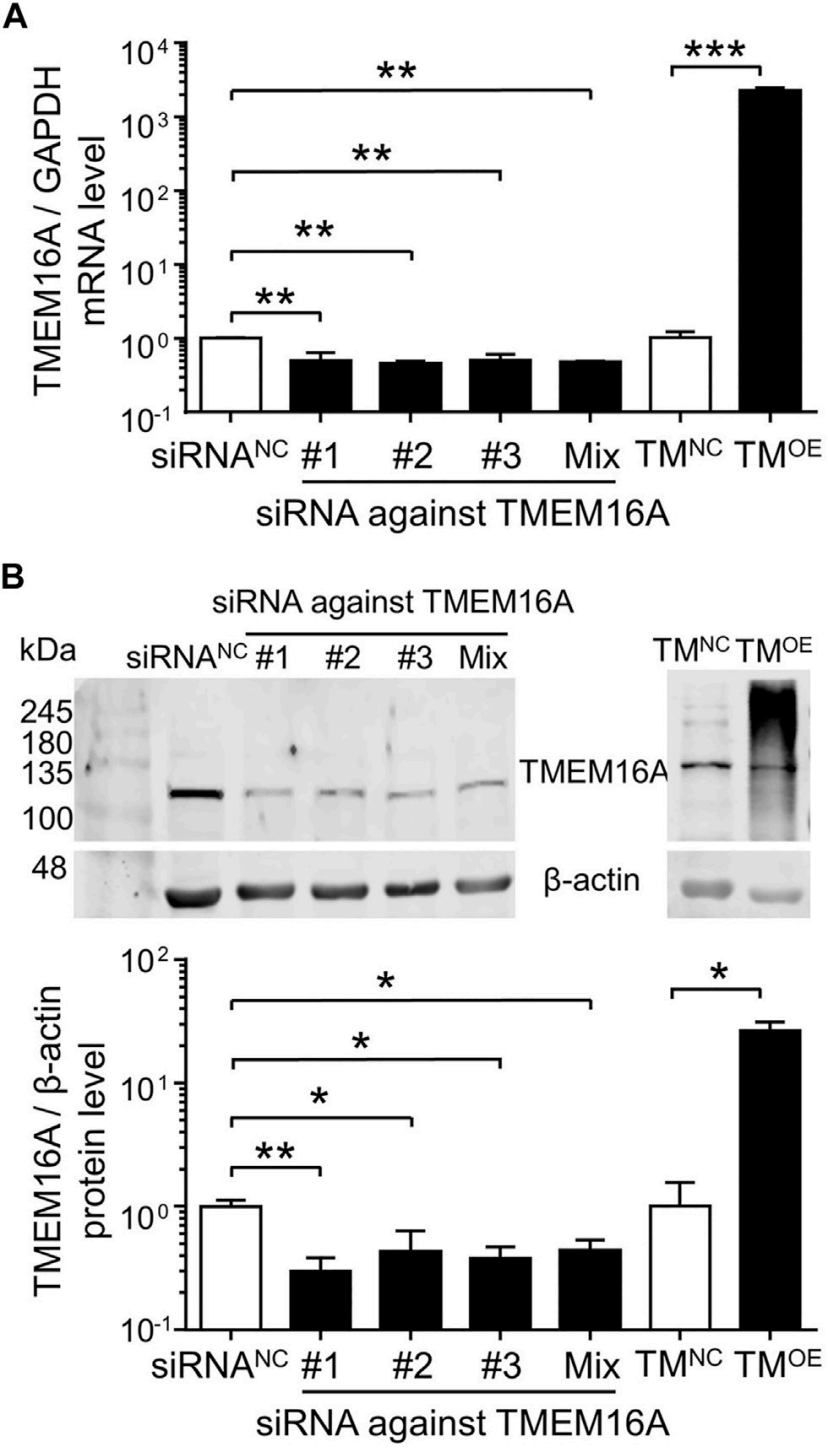
Molecular expression levels of TMEM16A in HUVECs. **(A)** TMEM16A mRNA expression level in HUVECs (n = 3–4). **(B)** Western blot results of TMEM16A protein expression level in HUVECs (*n* = 3–5). *p < 0.05, **p < 0.01 and ***p < 0.001, one-way ANOVA and unpaired *t*-test.

Equal proportion mixtures of three kinds of siRNA against TMEM16A were labeled as siRNA^Mix^. As shown in Figures 5A,B, siRNA against TMEM16A (#1, #2, #3 and Mix) transfection significantly decreased the TMEM16A mRNA and protein expression level, whereas TM^OE^ transfection significantly increased the TMEM16A molecular expression levels in HUVECs*.* Two clear bands appeared on TM^OE^ lane (Figure 5B) in western blot assay, the upper thicker band represented overexpressed mouse TMEM16A protein induced by lentivirus, the down thinner band represented endogenous human TMEM16A protein expressed in HUVECs.

### 3.7 Effect of TMEM16A on Migration in HUVECs

**FIGURE 6:**
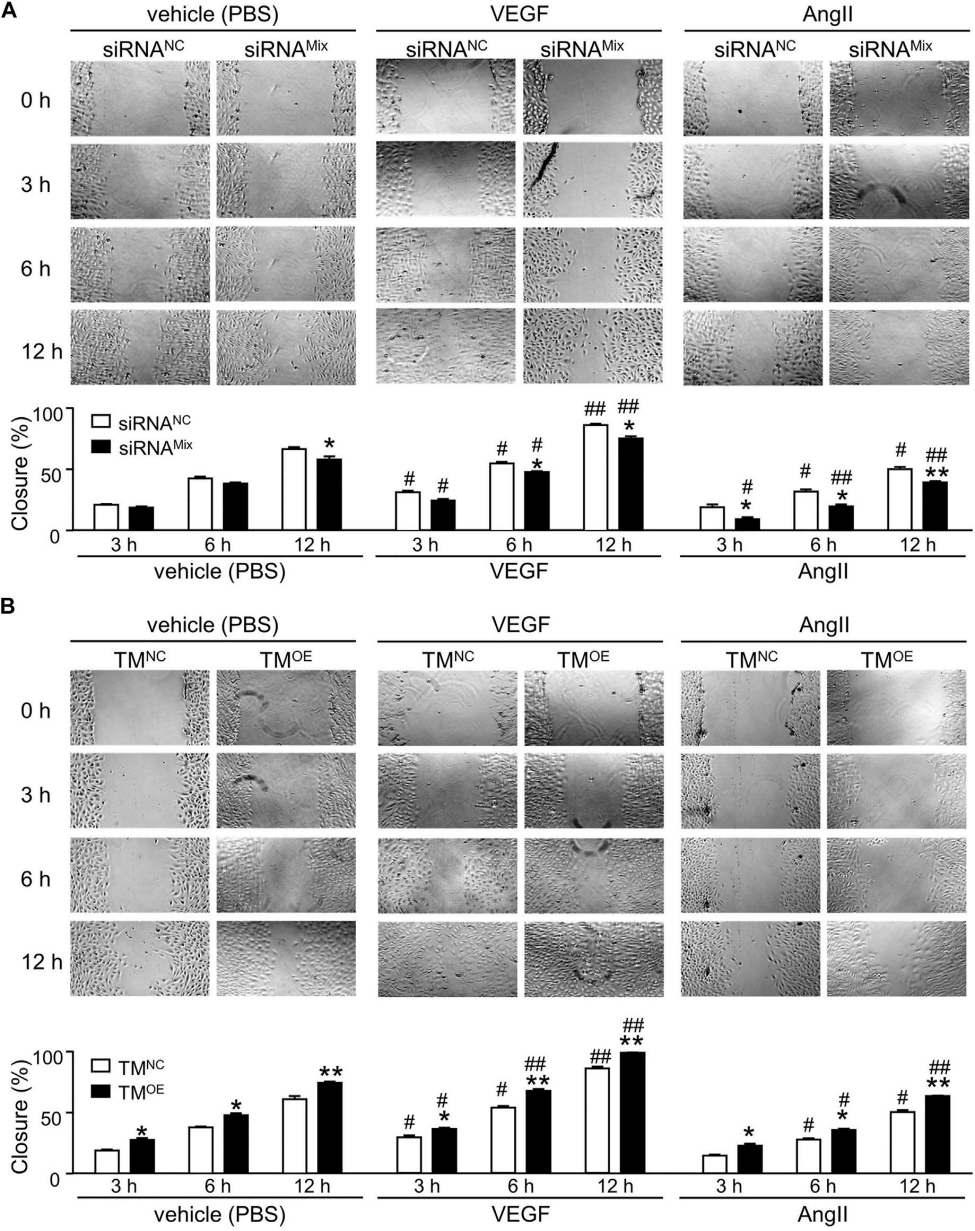
Effect of TMEM16A on migration in HUVECs. **(A)** Wound healing assay results of HUVECs transfected with siRNA^NC^ or siRNA^Mix^, and treated with vehicle (PBS), or VEGF (30 ng/mL), or AngII (1 μM) separately (*n* = 5—8 in each group per time point). **(B)** Wound healing assay results of HUVECs transfected with TM^NC^ or TM^OE^, and treated with vehicle (PBS), or VEGF (30 ng/mL), or AngII (1 μM) separately (n = 5 – 7 in each group per time point). *p < 0.05 and **p < 0.01 compared with siRNA^NC^ or TM^NC^ under the same treatment and at the same time point, #*p* < 0.05 and ##*p* < 0.01 compared with vehicle (PBS) in the same cell type and at the same time point, RM two-way ANOVA.

Knockdown of TMEM16A by siRNA^Mix^ transfection significantly decreased the wound closure percentage (Figure 6A, left), overexpression of TMEM16A by TM^OE^ transfection significantly increased the wound closure percentage (Figure 6B, left), suggesting that TMEM16A is a positive regulator of migration in HUVECs. Compared with vehicle (PBS), 30 ng/mL VEGF strongly promoted the wound healing progression in HUVECs (Figures 6A,B, left and middle). TMEM16A knockdown significantly decreased the migration promoting effect of VEGF (Figure 6A, middle), TMEM16A overexpression significantly increased the migration promoting effect of VEGF (Figure 6B, middle), suggesting that TMEM16A is a positive regulator of the migration promoting effect of VEGF in HUVECs. 1 μM AngII significantly inhibited the wound healing progression in HUVECs compared with vehicle (PBS) (Figures 6A,B, left and right). TMEM16A is a negative regulator of migration inhibiting effect of AngII in HUVECs since TMEM16A knockdown enhanced (Figure 6A, right) and TMEM16A overexpression attenuated (Figure 6B, right) the migration inhibiting effect of AngII.

### 3.8 Effect of TMEM16A on Angiogenesis in HUVECs

**FIGURE 7:**
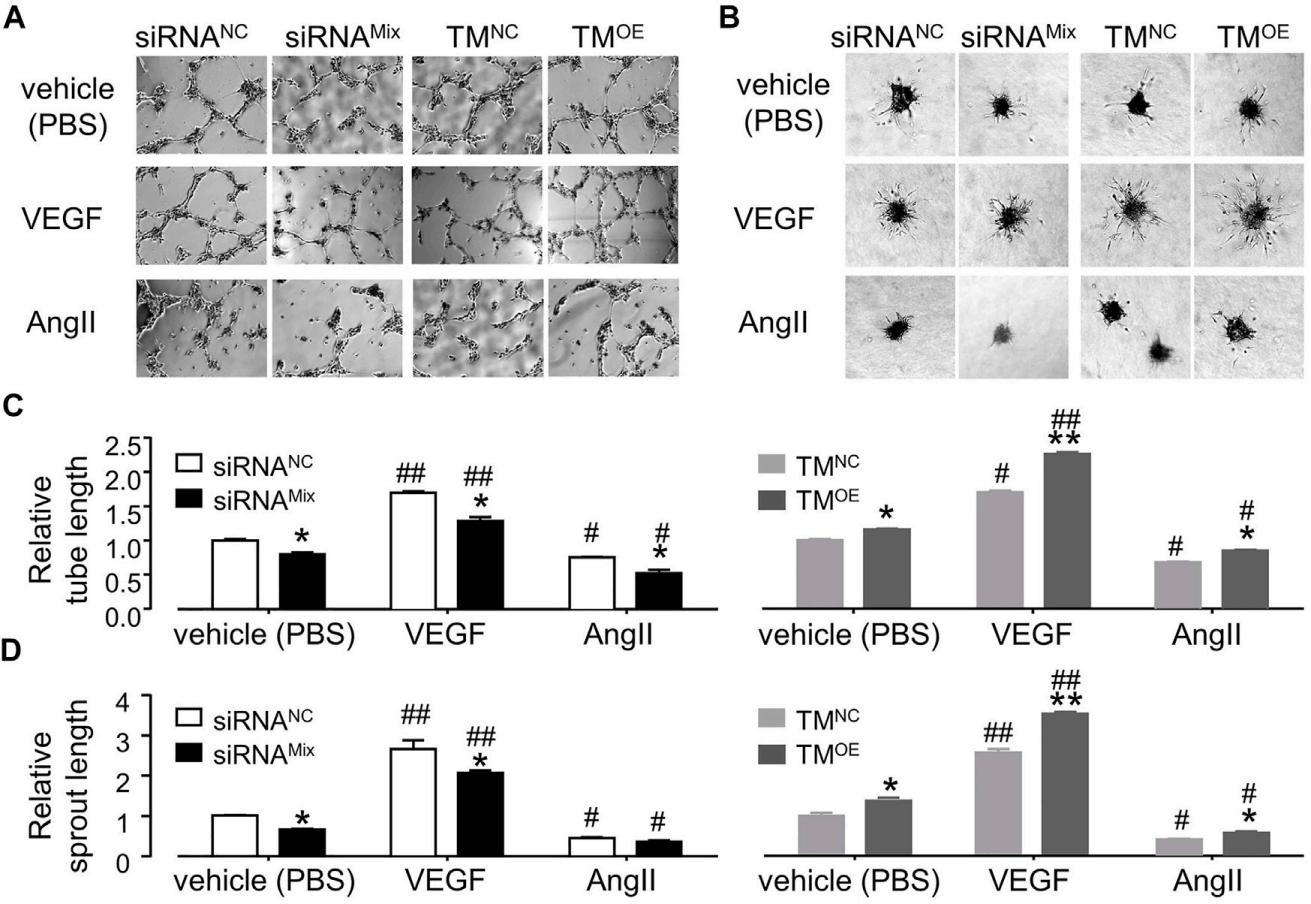
Effect of TMEM16A on angiogenesis in HUVECs. **(A,C)** Tube formation assay results of HUVECs transfected with siRNA^NC^ or siRNA^Mix^, or TM^NC^, or TM^OE^, and treated with vehicle (PBS), or VEGF (30 ng/mL), or AngII (1 μM) separately (n = 3–9). **(B,D)** Endothelial cell spheroids sprouting assay results of HUVECs transfected with siRNA^NC^ or siRNA^Mix^, or TM^NC^, or TM^OE^, and treated with vehicle (PBS), or VEGF (30 ng/mL), or AngII (1 μM) separately (*n* = 3–5). *p < 0.05 compared with siRNA^NC^ or TM^NC^ under the same treatment, #*p* < 0.05 and ##*p* < 0.01 compared with vehicle (PBS) in the same cell type, two-way ANOVA.

As shown in Figures 7A–D, TMEM16A knockdown significantly decreased the total branches length in tube formation assay and the total sprouts length in cell spheroids sprouting assay, TMEM16A overexpression exhibited opposite effects, suggesting that TMEM16A is a positive regulator of angiogenesis in HUVECs. 30 ng/mL VEGF significantly promoted angiogenesis, whereas 1 μM AngII significantly inhibited angiogenesis according to the results of both tube formation assay and cell spheroids sprouting assay. TMEM16A knockdown significantly decreased and TMEM16A overexpression significantly increased the pro-angiogenesis effect of VEGF, suggesting that TMEM16A is a positive regulator of pro-angiogenesis function of VEGF in HUVECs. TMEM16A knockdown significantly enhanced and TMEM16A overexpression significantly attenuated the anti-angiogenesis effect of AngII, suggesting that TMEM16A is a negative regulator of anti-angiogenesis function of AngII in HUVECs.

## 4 Discussion

The properties of I_Cl.Ca_ and the possible roles of I_Cl.Ca_ in normal or diseased hearts have been studied for more than 30 years since I_Cl.Ca_ was firstly recorded in canine ventricular myocytes (Tseng and Hoffman, 1989). Besides the low-amplitude of I_Cl.Ca_ compared with other currents in cardiac cells, some factors also increase the difficulties of studying the role of I_Cl.Ca_ in cardiac physiology and pathophysiology. In whole-cell current recordings of cardiac cells, I_Cl.Ca_ is usually elicited by depolarization-activated Ca^2+^ influx *via* voltage-gated Ca^2+^ channels and Ca^2+^ release from SR instead of by a fixed concentration of Ca^2+^ dialyzed from pipette solution, for sake of being similar to the trigger conditions *in vivo*. Cl^−^ channel inhibitors such as niflumic acid (NFA), diisothiocyanato-stilbene-2,2′-disulphonic acid (DIDS) and 9-AC were commonly used to separate I_Cl.Ca_ from overlapped inwardly I_Ca_ (Tseng and Hoffman, 1989; Zygmunt, 1994; Verkerk et al., 2000; Verkerk et al., 2004; Pu et al., 2006). However, NFA and DIDS were proved not only inhibit I_Cl.Ca_ but also inhibit currents induced by other Cl^−^ channels. NFA and 9-AC activate Ca^2+^-activated K^+^ currents. NFA and 9-AC even enhances I_Cl.Ca_ under certain conditions (Greenwood and Leblanc, 2007; Schroeder et al., 2008). In 2008, TMEM16A was identified as a CACC, several TMEM16A specific pharmacological inhibitors were reported since then. Among them T16A_inh_-A01 was found specifically and efficiently inhibiting I_TMEM16A_ in cardiomyocytes, salivary gland cells and vascular smooth muscle cells (Namkung et al., 2011a; Sanders et al., 2014; Ye et al., 2015), though T16A_inh_-A01 also displayed poor selectivity for TMEM16A in vascular tissue (Boedtkjer et al., 2015). Pore-targeting TMEM16A antibodies were considered to inhibit I_TMEM16A_ well (Thomas-Gatewood et al., 2011; Davis et al., 2013; Ye et al., 2015), but large-scale application of antibodies costs a lot. In this study, the inhibition of I_TMEM16A_ by T16A_inh_-A01 or by TMEM16A antibody was insignificantly different, so T16A_inh_-A01 was used instead of antibody in the following experiments.

We found that neither TMEM16A molecular expression levels in LV nor whole-cell I_TMEM16A_ density in LVMs varied significantly during the development of mice CH and HF induced by pressure-overload. Whole-cell I_TMEM16A_ densities among normal LVMs, hypertrophied LVMs and failing LVMs were similar, one possible reason is the similar TMEM16A molecular expression levels in these cells. Moreover, changes of Ca^2+^ homeostasis in hypertrophied and failing cardiomyocytes may not appear in whole-cell patch-clamp experiments *in vitro*, since we recorded current by one depolarizing stimulus each time on one cell, and Houser et al. reviewed that Ca^2+^ transients are similar in non-failing and failing myocytes at very slow frequencies of stimulation (Houser et al., 2000). Prolongation of APD in hypertrophied and failing LVMs was observed; inhibition of I_TMEM16A_ unchanged APD in any cardiomyocytes in this study. Verkerk et al. found that HF per se did not alter I_Cl.Ca_ density in rabbit (Verkerk et al., 2001), Pu et al. reported that I_Cl.Ca_ density decreased significantly in failing canine cardiomyocytes, which may contribute to the prolongation of APD in failing heart (Pu et al., 2006). Different usages of cell species and experimental methods may explain the different observations.

We have not got evidence that TMEM16A contributes directly to remodeling of the myocardium during pressure-overload, but we wound not simply assert that TMEM16A is meaningless in CH or HF. Just as people cannot deny the crucial significance of L-type Ca^2+^ channels and SR calcium pump (Ca-ATPase) in Ca^2+^ homeostasis of heart, though many reports supported that the molecular expression levels of these two proteins, I_Ca_ densities induced by L-type Ca^2+^ channels and Ca^2+^ uptake rates of SR Ca-ATPase are all changeless in hypertrophied and failing cardiomyocytes with impaired Ca^2+^ homeostasis (Houser et al., 2000; Pitt et al., 2006). Functional proteins always work together to modulate metabolism in cells.

The heart responds to stress such as pressure-overload with an increase in cardiac muscle mass (hypertrophic myocardium remodeling) as well as changes on angiogenesis. We found that TMEM16A is a positive regulator of migration and angiogenesis in HUVECs both under normal condition and under simulated stress. Similar and opposite opinions exist in the previous publications. In mouse and bovine brain capillary ECs, T16A_inh_-A01 reduced cell viability in a concentration-dependent manner. Either T16A_inh_-A01 or TMEM16A knockdown also curtailed cell proliferation and migration, suggesting that TMEM16A is a positive regulator of proliferation and migration (Suzuki et al., 2020). In human aortic ECs, TMEM16A is a negative regulator of proliferation, migration and angiogenesis. TMEM16A inhibition by cholesterol contributes to endothelial dysfunction (Ma et al., 2021). In HUVECs, TMEM16A is a positive regulator of reactive oxygen species generation, which induces endothelial dysfunction. Studies on endothelial-specific TMEM16A modification mice showed that TMEM16A is a positive regulator of endothelial dysfunction (Ma et al., 2017). In cardiac vascular ECs of neonatal mice, hypoxia-induced increase in proliferation rate was not affected by T16A_inh_-A01 (Wu et al., 2014). Among hunderds of N-aroylaminothiazole chemical compounds, the most potent TMEM16A activator was named “E_act_” (Namkung et al., 2011b). In rat lung microvascular ECs, hypoxia-induced increase in proliferation rate was not affected by TMEM16A knockdown, but activation of TMEM16A by E_act_ caused apoptosis, TMEM16A knockdown blocked the effects of E_act_. E_act_ affected insignificantly in normal human pulmonary arterial endothelial cells (PAECs), whereas in idiopathic pulmonary arterial hypertension PAECs, E_act_ enhanced apoptosis which can be attenuated by DIDS. DIDS led to no changes in normal or diseased PAECs (Ayed et al., 2018). TMEM16A inhibitor CACC_inh_-A01 attenuated brain infarct size, improved neurological outcomes and lowered blood-brain barrier permeability after ischemic stroke in mice (Liu et al., 2019). In sum, the contributions of TMEM16A in endothelial dysfunction, angiogenesis, migration, proliferation and apoptosis are not always same in ECs. Coincidentally, TMEM16A doesn’t affect, or positively regulates, or negatively regulates the migration and proliferation of different types of tumor cells (Oh and Jung, 2016). It’s not a rare situation that TMEM16A behaves diversely in different types of cells or under different conditions.

Over the past decades, lots of efforts have been made to modulate angiogenesis as a therapeutic strategy to either promote revascularization of ischemic tissues or inhibit angiogenesis in cancer and retinopathies. For instance, several VEGF inhibitors have been approved for the treatment of cancer and the neovascular form of age-related macular degeneration (Ferrara et al., 2007). However, no agents that promote angiogenesis have shown sufficient efficacy to be approved, despite considerable progress on growth factors such as VEGF and FGF-2 in the management of ischemic cardiovascular disease in initial exploration of therapeutic angiogenesis in animal models and Phase one clinical trials (Nowak-Sliwinska et al., 2018; Slater et al., 2019). AngII is involved in anti-angiogenesis under certain circumstances, for example, AngII treatment significantly impaired angiogenetic responses in cardiac microvascular endothelial cells (Belabbas et al., 2008; Guan et al., 2013). Since we found that modification of TMEM16A expression affects migration and angiogenesis in HUVECs, and changing of TMEM16A expression can also influence the function of VEGF and AngII regarding the migration and angiogenesis, TMEM16A may become a new potential target on therapy of pathological angiogenesis forced diseases and ischemic diseases, when co-working with or without other agents.

Overall, this study concludes that TMEM16A exhibits insignificant role in myocardium remodeling of heart during pressure-overload in mice. TMEM16A is a positive regulator of angiogenesis both under normal condition and under simulated stress *in vitro*. TMEM16A may take part in angiogenesis instead of myocardium remodeling in heart under stress. These suggest that TMEM16A may become a new target for upregulation of angiogenesis in order to regrow blood vessels in ischemic disorders such as ischemic heart disease.

## Data Availability

The raw data supporting the conclusion of this article will be made available by the authors, without undue reservation.
