# Supplementary figures and images for: TMEM16A Plays an Insignificant Role in Myocardium Remodeling but May Promote Angiogenesis of Heart During Pressure-overload
Source: Front Physiol. 2022 May 31;13:897619. doi: 10.3389/fphys.2022.897619 (PMC9194855; doi:10.3389/fphys.2022.897619)

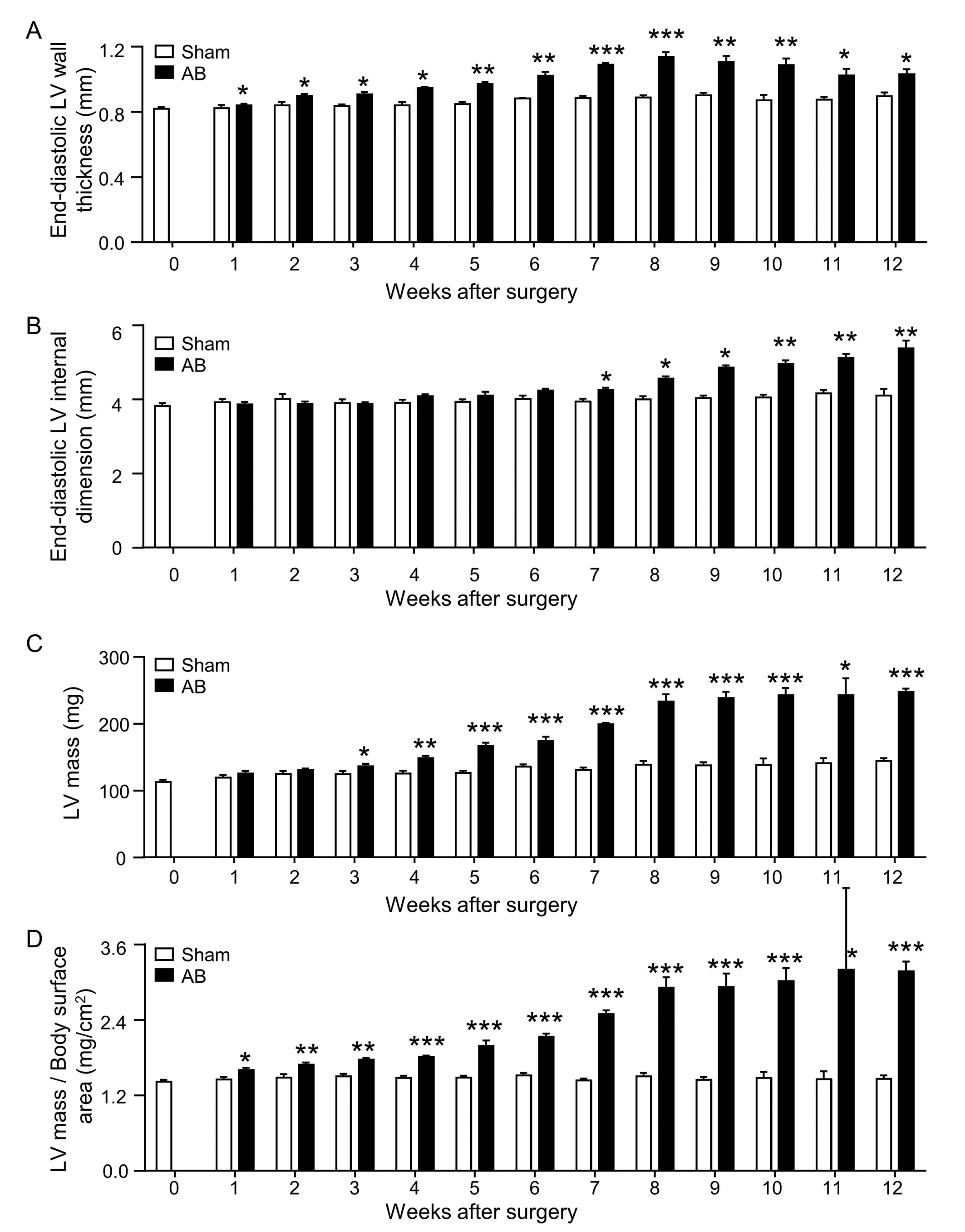

Supplement: Supplementary file 1 [file Image1.JPEG]

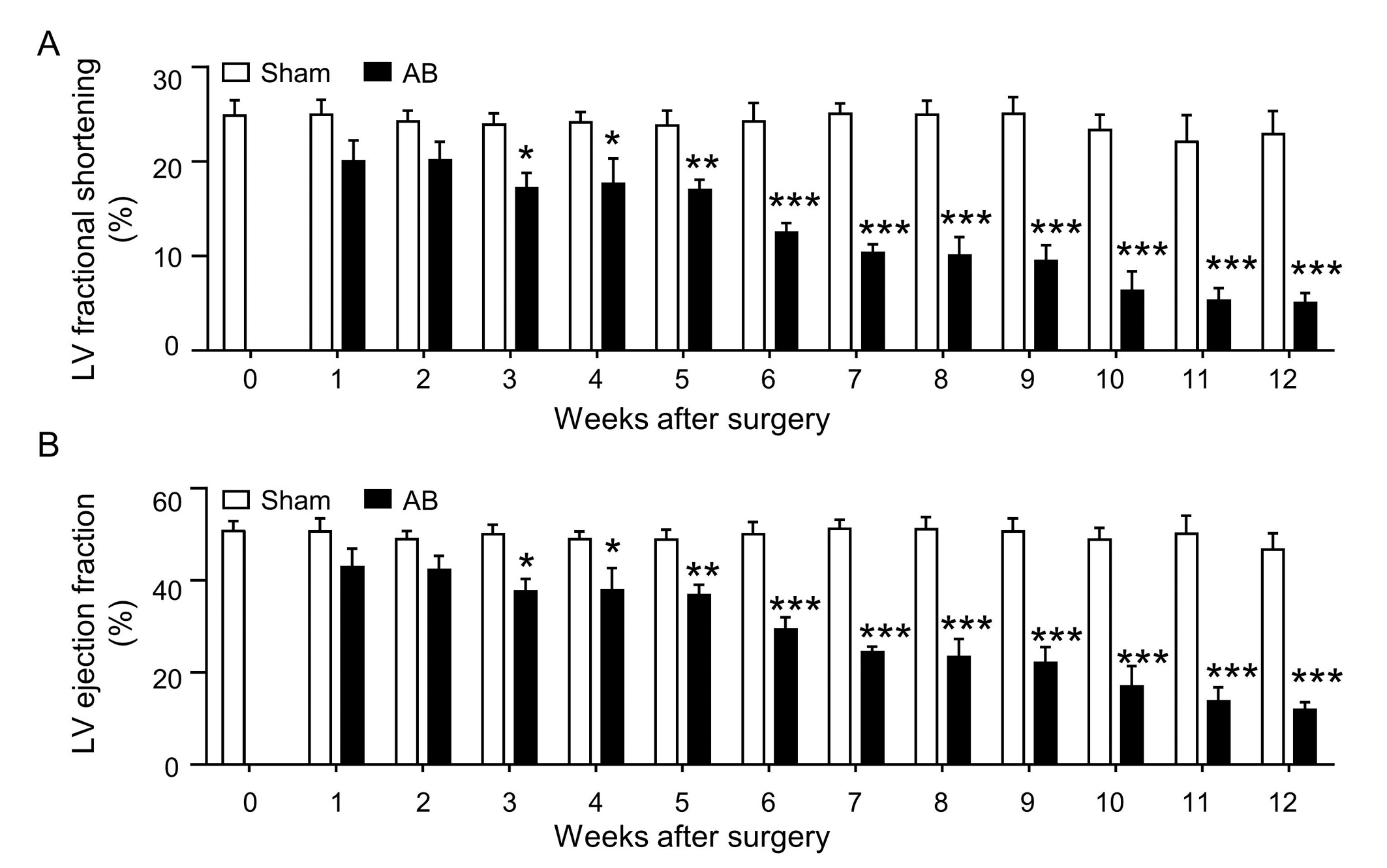

Supplement: Supplementary file 2 [file Image2.JPEG]
