# Supplementary material for: TMEM16A Plays an Insignificant Role in Myocardium Remodeling but May Promote Angiogenesis of Heart During Pressure-overload
Source: Front Physiol. 2022 May 31;13:897619. doi: 10.3389/fphys.2022.897619 (PMC9194855; doi:10.3389/fphys.2022.897619)
Supplement: Supplementary file 3 [file DataSheet1.docx]

Supplementary Material

**Supplementary Figure Legends**

**FIGURE S1 | (A)** End-diastolic LV wall thickness (n = 6 – 18 mice in each group per time point). **(B)** End-diastolic LV internal dimension (n = 6 – 18 mice in each group per time point). **(C)** LV mass (n = 6 – 18 mice in each group per time point ). **(D)** LV mass / Body surface area (n = 6 – 18 mice in each group per time point). **P* < 0.05, ***P* < 0.01 and ****P* < 0.001 compared with sham at the same time point, RM two-way ANOVA.

**FIGURE S2 | (A)** LV fractional shortening (n = 6 – 18 mice in each group per time point). **(B)** LV ejection fraction (n = 6 – 18 mice in each group per time point). **P* < 0.05, ***P* < 0.01 and ****P* < 0.001 compared with sham at the same time point, RM two-way ANOVA.
